# Unveiling the influence of global innovation networks on corporate innovation: evidence from the international semiconductor industry

**DOI:** 10.1038/s41598-024-61511-7

**Published:** 2024-05-14

**Authors:** Yingna Wu, Liang Ding, Na Li, Xin Yu

**Affiliations:** 1 School of Business Administration, Zhongnan University of Economics and Law, Wuhan, China; 2 Applied Economic Research Center, Jiangxi Institute of Fashion Technology, Nanchang, China; 3 School of International Trade and Economics, Jiangxi University of Finance and Economics, Nanchang, China

**Keywords:** Global innovation networks, Innovation performance, Centrality, Structural hole, R&D, Risk factors, Engineering

## Abstract

In this study, we investigate the influence of global innovation networks (GINs) on the innovation output of semiconductor firms. Utilizing negative binomial regression and network analysis, we assess how network positions, specifically degree, betweenness, and closeness centrality, affect firms’ innovation performance, revealing significant positive impacts. Moreover, our results identify a positive U-shaped relationship between structural holes in GINs and innovation performance, suggesting that while moderate network engagement aids innovation, too much can be detrimental. This research provides key insights into optimizing GIN participation for better innovation results in the competitive semiconductor sector.

## Introduction

As economic globalization and internationalization of business cooperation continue to expand, factor resources are being redistributed worldwide. In this context, closed innovation is no longer a suitable development strategy for enterprises or countries. In the face of asymmetric market information and an unstable innovation environment, seeking external resources to reduce R&D risks has become the first choice of enterprises. Through open collaborative innovation modes, enterprises can seize strategic high points in science and technology development and even dominate the industrial development landscape.

The semiconductor industry is characterized by innovation, and its innovative output is closely related to the future development trend of various products such as chips, crystals, hardware, and integrated circuits, which are the "lifeblood" of the sustainable development of enterprises and countries. As a high-technology threshold industry, the semiconductor industry faces increasingly fierce international competition, higher technological complexity, and faster technology upgrading. The networking and globalization of enterprises in the semiconductor industry are becoming more apparent, as companies are unable to complete complex technological research solely through their resources and funding, leading to an increased reliance on external innovation resources and information. Frequent patent citation activities between enterprises reflect the industry's networking and globalization. Additionally, multinational companies in the semiconductor industry allocate resources globally, and technology, information, knowledge, and other resources flow across borders between companies.

While global innovation networks (GINs) are recognized for their benefits across industries, this paper focuses on their distinct role within the semiconductor sector, highlighting how they mitigate risks and costs amidst market information asymmetry. Our approach addresses an underexplored area, emphasizing the sector-specific advantages of GINs in bolstering international competitiveness. This paper aims to investigate the impact of such networks on corporate innovation performance, particularly in the context of the international semiconductor industry. The following sections will detail the mechanisms through which GIN embedding affects semiconductor companies and propose a set of hypotheses for empirical testing.

The remainder of this paper is structured as follows: Sect. “Literature review” provides literature review; Sect. “The mechanism and hypothesis” discusses the mechanism and proposes hypotheses; Sect. “Model and data” focuses on the models and data description. We discuss the findings and results in Sect. “Empirical analysis”. The major propositions and policy implications are given as a conclusion in Sect. “Conclusions”.

## Literature review

Although the relevance of Global Innovation Networks (GINs) is well-documented, this paper identifies a critical gap: the lack of in-depth studies focusing on the semiconductor industry's unique context. This review systematically addresses this oversight, setting a foundation for examining GINs' impact from a semiconductor-centric perspective. Ernst^1^ first proposed the concept of the "Global Innovation Network." By studying the inherent relationship between regional production and global production, Ernst^1^ argued that the GIN is a network form that integrates decentralized engineering applications, product development, and R&D activities across organizational boundaries and regional boundaries. Kollmann^2^ pointed out that the GIN is an innovation model that emerges when enterprises shift from closed innovation to open innovation in the context of economic globalization. Furthermore, Sachwald^3^ explores the strategic locational decisions within GINs, particularly focusing on Europe's positioning, which sheds light on the geographical dimension of innovation networks that complements the global perspective brought forward by Cooke^4^. Cooke^4^ compared and analyzed the GIN with the global innovation system, highlighting that the GIN is not a general virtual network but a special structural organization that also includes suppliers, companies, and their competitors contained in the industrial chain.

Ale^5^ proposed that open innovation is relative to "closed innovation." The social environment that nurtures open innovation mainly includes three characteristics: first, the rapid renewal of knowledge; second, the wide distribution of knowledge; and third, cross-regional cooperation is becoming more and more apparent. Expanding on the concept of open innovation, Malecki^6^ discusses the symbiotic relationship between local entrepreneurial ecosystems and GINs, emphasizing the integration of global and local networks in fostering comprehensive innovation capabilities. Vanhaverbeke^7^ believes that open innovation means that the product development process within the enterprise is not limited to the participation of internal personnel, and that valuable technologies or products that can be developed outside the enterprise can also be used for their own use. The role of learning within GINs, as highlighted by Shayan et al.^8^, provides an exemplary model of self-organized innovation networks, illustrating how embedded case studies can inform the design and functioning of these networks for enhanced innovative output.

GINs can help address the deficiencies of internal innovation systems. The flow of knowledge, information, and resources within GINs can accelerate learning and the acquisition of new knowledge and resources, prevent the risk of internal research and development failure, reduce high costs associated with R&D, and ultimately improve the innovation capability and performance of enterprises^9,10^. In a similar vein, Cano-Kollmann et al.^11^ delve into the organizational and individual layers of GINs, demonstrating how these networks serve as platforms for decentralized innovation activities across borders. This perspective complements Ernst's^1^ foundational view by providing a nuanced understanding of how personal and organizational interactions within GINs drive the cross-regional innovation processes. Liu and Lü^12^ conducted a questionnaire survey on 267 startups in the Yangtze River Delta in China and created a knowledge cooperation network using Likert 5-scale scoring measurement. Additionally, the work by Hu et al.^13^ on the spatiotemporal evolution of GINs and China's shifting role enriches our understanding of the dynamic nature of global innovation collaborations, echoing the importance of structural position highlighted by Lü et al.^14^. Their research shows that the structural embedment of enterprises in the network has a positive impact on the innovation of enterprises. However, relational embedding has no significant effect on innovation performance. Gao et al.^15^ built an industry-university-research cooperative innovation network by connecting the patent data of enterprises and found that the clustering coefficient of the cooperative network had a positive effect on the innovation performance of enterprises in the sampling period. De Prato and Nepelski^16^ further elaborate on the intricate patterns of international co-inventions, underscoring the value of global technological collaborations. Their findings complement the discussions on cooperative innovation networks by providing empirical evidence from the analysis of global co-inventions.

However, on the other hand, Zhang^17^ analyzed the patent data of semiconductor companies in China and found that when the density of cooperation networks within an organization increases to a certain extent, the spread of explicit knowledge and tacit knowledge will be inhibited, and the information homogenization will be serious, hindering the proposal of innovative ideas, and thus not conducive to the generation of progressive inventions and breakthrough innovations in enterprises. Yao and Gong^18^ used the patent applicants of 165 listed enterprises from 2007 to 2018 to establish a network and found that the knowledge network structure hole of enterprises positively affects exploitative innovation performance through the acquisition of heterogeneous information, knowledge, and resources but inhibits exploratory innovation.

In the study of the impact of GIN embeddedness, scholars typically measure the subject's embeddedness in the network through network location. Therefore, most scholars use centrality and structural hole indicators to investigate the impact of network embeddedness. The different positions of enterprises in the GIN can affect their opportunities and conditions for accessing new technologies, knowledge, and innovation resources^14^. Therefore, a firm's position in the GIN is a critical factor affecting its technological innovation capability. Ho and Chiu^19^ found that firms at the center of the knowledge network outperformed firms at the edges of the network in terms of financial performance by establishing a knowledge flow network among the top 30 semiconductor firms in the world, as measured by centrality. Furthermore, the denser the innovation network, the more frequently external knowledge, technology, information, and other resources are disseminated among the nodes in the network. The innovation subjects in the center position of the network have more access to external information, resources, and social capital^20–23^. The node in the structural hole of the network is in the intermediary position, which enables it to access and control more key knowledge sources. The node in this position has strong control and can use it to acquire, absorb, and utilize resources. However, when the number of structural holes in the network exceeds a certain limit, the network becomes too sparse due to the number of structural holes, which can eventually bring adverse effects to the enterprise^24^.

Scholars have conducted systematic analysis and research on the influencing factors of innovation performance and the influence of global innovation networks. These research theories and results not only provide research ideas and directions for international business research but also establish a solid foundation for the research in this paper. However, there is limited literature that studies the impact of GIN on enterprises from the perspective of enterprises, especially for semiconductor companies. Scholars often establish cooperative innovation networks through cooperative patents between enterprises, while studies on the global innovation network from the perspective of patent citation activities are rare.

Therefore, this paper takes international semiconductor companies as the research object, and analyzes and describes the characteristics of the GIN formed by these semiconductor companies as a whole. It conducts a mechanism analysis and puts forward hypotheses on how GIN embedding affects the innovation performance of selected semiconductor companies. Our contribution is to study the impact of the degree of embedding of semiconductor companies in the global innovation network on enterprise performance from the perspective of enterprises.

Furthermore, most previous studies in the field of international business have utilized subjective questionnaires, interviews, or patent cooperation activities as a way to build social networks. This method is relatively single and subjective and often has limited research sample sizes, typically within a certain industry in a specific country or region. Our paper enriches the literature by building a GIN using patent citation activities and selecting 20 international semiconductor companies, accounting for 81% of the global market share as samples. The empirical analysis underscores a novel finding: within the semiconductor industry, key network positions such as degree centrality, betweenness centrality, and closeness centrality exert a substantial positive influence on innovation performance. This sector-specific insight marks a significant contribution to the extant body of GIN literature. Structural holes in the GIN have a positive "U" shaped relationship with enterprise's innovation performance.

## The mechanism and hypothesis

### The impact of structure hole in GIN on enterprise innovation performance

The position of a semiconductor company in the global innovation network can be represented by the concept of a "structural hole." The location of nodes in the network has a significant impact on their ease of accessing social resources, knowledge, and technologies. Nodes in advantageous positions are better equipped to obtain and control resources, knowledge, technology, and information compared to other nodes in the network. These nodes act as intermediaries, brokers, or third parties, transferring and disseminating information, knowledge, and resources to other nodes^24^.

Nodes in the position of a "structural hole" have many advantages. First, they have access to more resources, including invisible knowledge and scarce resources, and can learn and utilize knowledge from multiple channels, improving the competitiveness and knowledge creativity of enterprises. Second, as intermediaries, they have the power to selectively retain and disseminate knowledge, information, and technology while transmitting them^25^. They can use their negotiation ability to obtain information, knowledge, and resources that could not be obtained directly, enhancing the competitiveness, importance, and innovation of their enterprises. However, Yao and Gong^18^ suggest that the structure of corporate knowledge networks can negatively affect the performance of exploratory innovation.

In this study, we measure the structural hole using the index of the degree of restriction. A lower degree of restriction indicates that an enterprise has lower restrictions in the global innovation network and can access more differentiated innovation resources. However, due to the high-tech density of the semiconductor industry, companies have a high monopoly on knowledge, and cooperation and exchanges between companies are less frequent. Acquiring heterogeneous resources, information, and knowledge from other enterprises requires a significant investment of time and resources, including filtering low-quality, repetitive, and redundant knowledge and information. This can initially hinder enterprise innovation after obtaining heterogeneous resources. However, as companies gain more learning experience, they can absorb, use, and acquire heterogeneous resources, information, and knowledge more efficiently, enhancing their R&D capabilities and realizing technological innovation.

In this study, the global innovation network (GIN) position of a semiconductor company is characterized using degree centrality, betweenness centrality, closeness centrality, and structural holes. Degree centrality indicates the direct connections a company holds, influencing its resource accessibility. Betweenness centrality reflects a company's intermediary role, impacting information and resource control. Closeness centrality measures how quickly a company can access network-wide information, affecting its responsiveness to innovation. The concept of 'structural hole' signifies gaps between non-redundant contacts, allowing companies to bridge diverse groups and foster innovation by combining distinct knowledge sets. While detailed discussions on these indicators will follow, their collective impact is central to understanding a firm's innovation potential within the GIN.

Based on these insights, we propose the following hypothesis:

H1: The impact of structural changes in the network on the innovation performance of semiconductor companies shows a positive "U" shape.

### The Impact of degree centrality in GIN on enterprise innovation performance

Degree centrality, a measure of a node's connectivity within a network, signifies the direct ties an enterprise maintains with others in the global innovation network (GIN). This metric is crucial for deciphering how a firm's central position in a network facilitates access to a variety of resources and information, pivotal for fostering innovation. Recent network theory suggests that a firm's strategic network position significantly boosts its innovative capabilities by enhancing the diversity of information flow and resources^26,27^.

Referring to Borgatti and Halgin^26^, we explore the notion that firms with high degree centrality are strategically placed to utilize their network ties for gaining competitive advantages. These firms are privy to unique information, cutting-edge technologies, and potential collaborative ventures, all essential ingredients for innovation. Additionally, the expedited flow of information among centrally located nodes markedly reduces the duration from ideation to market launch, a critical aspect for maintaining a competitive edge in dynamic industries like the semiconductor sector^28,29^.

Nonetheless, the pathway from degree centrality to innovation performance is not straightforward but is influenced by various factors, including a firm's absorptive capacity and the quality of its network ties. The nuanced nature of these relationships necessitates a thorough examination of the ties' characteristics and the firm's prowess in assimilating and utilizing new knowledge^30,31^.

Given these considerations, we propose the following hypothesis:

H2: The degree centrality within the GIN significantly and positively impacts the innovation performance of semiconductor enterprises, with the firm's absorptive capacity and the quality of its network ties serving as moderating factors.

### The impact of betweenness centrality in GIN on enterprise innovation performance

Betweenness centrality quantifies a node's role as an intermediary within a network, bridging the gap between disparate nodes. This crucial position empowers a firm to facilitate extensive connections, communications, and the transfer of knowledge across the network, thereby amplifying its influence. Nodes with high betweenness centrality are pivotal in the dissemination of information, knowledge, and resources, accumulating a wealth of insights from various sectors of the network. Such strategic positioning enables enterprises to wield control over the flow of knowledge, information, and resources within the GIN, capturing valuable inputs for innovation.

By acting as conduits in the GIN, firms not only gather diverse information and resources from other entities within the semiconductor industry but also leverage these assets to bolster their technological innovation capabilities. The integration, utilization, and absorption of both tangible and intangible resources circulating through these intermediaries underscore the firms' ability to innovate and adapt. Empirical research underscores the value of betweenness centrality for firms seeking to navigate and exploit the complex web of global innovation networks. Studies highlight how firms positioned as central intermediaries are more adept at identifying and capitalizing on new technological trends and market opportunities, directly contributing to their innovation output^32,33^.

Considering the dynamic interplay of information flow and resource exchange facilitated by betweenness centrality, we propose the following hypothesis:

H3: Betweenness centrality within the GIN significantly and positively impacts the innovation performance of semiconductor enterprises.

### The impact of closeness centrality in GIN on enterprise innovation performance

Closeness centrality is pivotal in understanding a node's efficiency in a network, measuring the average shortest path from a given node to all other nodes. This indicator is critical, as nodes with higher closeness centrality are not only in closer proximity to others but also possess enhanced relational ties and wield greater influence within the network. Studies such as those by Freeman^34^, who introduced the concept of centrality in social networks, highlight how such proximity facilitates swift and cost-effective access to diverse technological domains, thereby accelerating the flow of knowledge, social capital, and managerial insights. Moreover, Borgatti and Halgin^35^ further elucidate that this accelerated exchange is instrumental in the absorption and assimilation of innovative knowledge and resources, underlining the significance of closeness centrality in facilitating efficient network dynamics.

In the context of the semiconductor industry, where innovation technologies are closely guarded and exploratory innovation could risk information leakage, the role of closeness centrality becomes even more pronounced. Given the industry's heavy reliance on R&D investments, material resources, and specialized human capital to sustain technological leadership, a higher closeness centrality can offset some of these expenditures by streamlining the knowledge exploration process. This strategic positioning within the network not only augments the enterprise's rate of knowledge search but also catalyzes its competitive edge and innovation pace within the network. Empirical studies, such as the work of Zaheer and Bell^31^, have found evidence supporting the idea that firms with well-positioned network centrality exhibit enhanced innovation capabilities and performance. Further, Owen-Smith and Powell^36^ provide insights into how firms in high-tech sectors, including semiconductors, leverage network positions to gain access to novel information and resources, thus securing a more robust innovation performance.

The above is bolstered by the work of Singh and Phelps^37^, who demonstrate that the strategic advantage garnered from closeness centrality is not merely about reduced transactional distances but also about the quality and timeliness of knowledge integration.

Therefore, we advance the following hypothesis:

H4: Closeness centrality within the GIN significantly and positively influences the innovation performance of semiconductor enterprises, enhancing their competitiveness and rate of innovation within the network.

**Figure 1:**
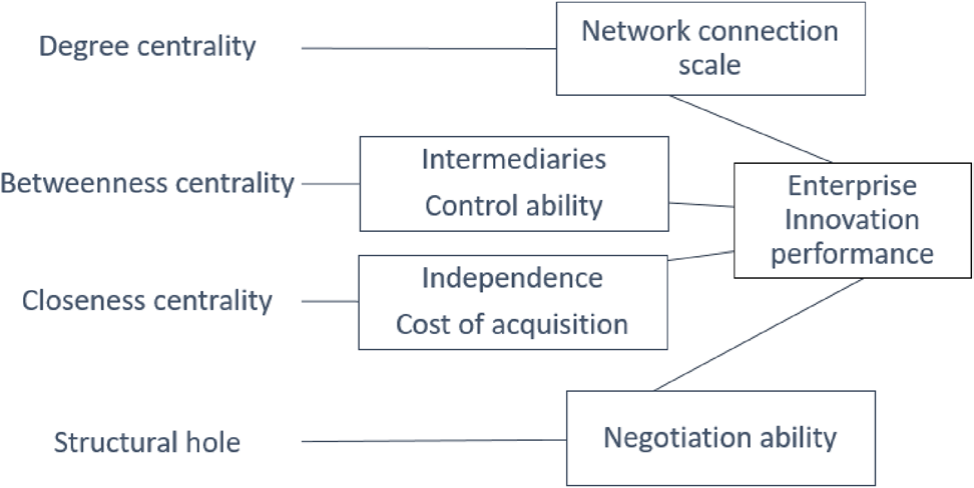
Theoretical relationship.

Based on the above analysis and assumptions, the theoretical model built in this paper is shown in Fig. 1:

## Model and data

### Sample and network

According to the statistics of the World Semiconductor Trade Association, 20 multinational companies accounted for 81% of the market share in 2018, namely: Samsung, Intel, Hynix, Micron, Qualcomm, Broadcom, Texas Instruments, NVIDIA, Toshiba, Western Digital, NXP, STMicroelectronics, Infineon, Sony, Apple, Huawei, MediaTek, Renesas Technologies, Micro Equipment, and Adeno Semiconductor Technologies Co., Ltd., as shown in Table 1. This indicates a high concentration of semiconductor suppliers, with the top five companies accounting for half of the industry's global revenue. The leading enterprises in the global semiconductor market are mainly Samsung, Intel, Micron, Hynix and other enterprises, which belong to the upper and midstream enterprises of the semiconductor industry chain and have great value-added space. The development trend and information resource flow of these 20 multinational enterprises fully reflect the development trend and characteristics of the entire industry. Therefore, this paper selects these 20 semiconductor multinational companies as the research object, and establishes global innovation networks among these 20 semiconductor companies based on the relationship between their semiconductor patent citations.

**Table 1:** Market shares of the top 20 semiconductor companies by sales revenue in 2018.

Rank	Enterprise	Market share	Rank	Enterprise	Market share
1	SAMSUNG	17%	11	STMicroelectronics	2%
2	Intel	15%	12	NXP	2%
3	Hynix	8%	13	Infineon	2%
4	Micron	6%	14	Sony	2%
5	Broadcom	4%	15	MediaTek	2%
6	Qualcomm	4%	16	Huawei	2%
7	Texas instruments	3%	17	Apple	2%
8	NVIDIA	2%	18	Renesas technologies	1%
9	Toshiba	2%	19	Ultra WEI equipment	1%
10	Western digital	2%	20	ADI semiconductor	1%

Source: World Semiconductor Trade Statistics Organization.

Patent citation cooperation networks are important carriers of technology cooperation and knowledge spillovers. The network can show the position of an enterprise in the network, the way of knowledge transfer and its efficiency, and can also identify core patents and promote future technological development. Some scholars have established an innovation network based on the cooperation patents or cooperation alliance relationships between enterprises. Referring to Ho and Chiu^19^, this paper establishes a global innovation network of semiconductor companies based on patent owners as nodes and the citation relationships between them. Therefore, this paper uses the patent citation relationship of the top 20 global semiconductor companies to establish a patent citation matrix of A20*20. After binarizing the matrix, a patent citation cooperation network diagram is formed using UCINET software.

### Regression model

The dependent variable in the regression model is the patent citation of the semiconductor enterprise, which is a non-negative integer. If a simple linear regression is used for this type of data, the parameter estimates obtained by the regression may be invalid, inconsistent, and biased. The most commonly used of the data is the Poisson model, but this model requires the assumption that the variance is equal to the expectation, that is, equal dispersion. By using descriptive statistical analysis of the data, we find that the variance of the number of semiconductor patent applications is much larger than the mean, showing the characteristics of excessive dispersion. The distribution characteristics of the data in this paper cannot meet the hypothesis requirements of the Poisson model, indicating that if the Poisson model is used for regression, the significance level of the empirical regression results will be overestimated. For such excessively scattered counting data, the negative binomial model is chosen, and the applicability of negative binomial regression model to patent data has been confirmed^38^. Therefore, according to the distribution characteristics of sample data in this paper, we choose the negative binomial regression model to conduct regression analysis on the data of international semiconductor enterprises and test the hypothesis.

The regression model for this study was developed as follows.

1 $${{\text{Patent}}}_{it}={\upbeta }_{0}+{\upbeta }_{1}{X}_{it}+{\upbeta }_{2}{{\text{age}}}_{it}+{\upbeta }_{3}{{\text{percent}}}_{it}+{\upbeta }_{4}{{\text{scale}}}_{it}+{{\text{u}}}_{it},$$

Where i represents the firm (i.e. the patentee), t represents the year, ${{\text{Patent}}}_{it}$ represents the amount of patents owned by firm i in year t; and ${X}_{it}$ represents the indicators of the firm's position in the international innovation network, which are ${{\text{degree}}}_{it}$, ${{\text{closeness}}}_{it}, {{\text{betweenness}}}_{it}$ and ${{\text{sh}}}_{it}$, where ${{\text{degree}}}_{it}$ represents degree centrality; ${{\text{closeness}}}_{it}$ represents closeness centrality; ${{\text{betweenness}}}_{it}$ represents betweenness centrality; ${{\text{sh}}}_{it}$ represents structural hole, which are four explanatory variables; ${{\text{age}}}_{it}, {{\text{percent}}}_{it}$ and ${{\text{scale}}}_{it}$ are control variables, which represent the age of the firm, the percentage of semiconductor patents of the firm, and the total asset size of semiconductor firms; ${\upbeta }_{2}$, ${\upbeta }_{3}$ and ${\upbeta }_{4}$ are the coefficients of the corresponding control variables; and ${{\text{u}}}_{it}$ represents the error term.

In addition, the specific regression model for the explanatory variable of structural holes is as follows.

2 $${{\text{Patent}}}_{it}={\upbeta }_{0}+{\upbeta }_{1}{sh}_{it}+{\upbeta }_{2}{sh}_{it}^{2}+{\upbeta }_{3}{{\text{age}}}_{it}+{\upbeta }_{4}{{\text{percent}}}_{it}+{\upbeta }_{5}{{\text{scale}}}_{it}+{{\text{u}}}_{it}.$$

### Data description

This study collects the patent data of enterprises from the United States Patent Database (USPTO). Due to the quality of database and the impact of patents needs at least 5 years, we set the sampling period from 2014 to 2018. The reference data is about 350,000 related patents. The steps of data cleaning and sorting are as following: First, the patent number and the enterprise information referenced by the patent are reserved; Second, duplicate information is removed from the retained field information; Thirdly, the collected reference data of the patentee is converted into an adjacency matrix, that is, a 20*20 adjacency matrix is established for each year. From 2014 to 2018, the number of patent citations between 20 patentees per year is counted separately, and if the number of patent citations between patentees is 1, it is recorded as 1, and if the number of patent citations between patentees is N, then recorded as N. Due to the need of analysis, the established numerical matrix is binarized.

In addition, the age of the company and the total assets of the company are obtained by searching the information on the company's official website, financial statements and the American Stock Exchange. This paper finally obtained the balance panel data of 20 enterprises from 2014 to 2018.

To measure the innovation performance of international semiconductor enterprises, the number of semiconductor patent applications per year is used (*Patents*) as dependent variable.

The degree centrality (*DC*) refers to the size of the direct connection of patentee i in the GIN network in the network. The calculation formula is as follows:

3 $${DC}_{it}=\sum_{j}^{n}{x}_{ij}/({\text{n}}-1),$$

where ${DC}_{i}$ represents the degree centrality of the patentee i; j represents the other patentees in the GIN network; n indicates the total number of patentees in the network. ${x}_{ij}$=1 when patentee i is directly connected to patentee j, otherwise 0.

Betweenness centrality (*BC*) refers to the extent to which a patentee is located in the center of other patentees, which can directly measure the extent to which this patentee controls the communication of other patentees. The calculation formula is:

4 $${BC}_{it}=\sum_{j}^{n}\sum_{k}^{n}{b}_{jk}\left(i\right)/({n}^{2}-3n+2),$$

j ≠ i ≠ k and j < k.

Where ${b}_{jk}(i)={g}_{jk}(i)/{g}_{jk}$, ${g}_{jk}(i)$ represents the number of shortest paths between enterprises j and k through patentee I; ${g}_{jk}$ represents the number of shortest paths between enterprises j and k; n is the total number of patentees in the network.

Closeness centrality (*CC*) refers to the proximity between a patentee and other patentees, which is calculated as the sum of the lengths of the shortest paths between the patentee and all other patentees in the network. The calculation formula is as follows:

5 $${CC}_{RPi}^{-1}=\sum_{j=1}^{n}{d}_{ij}/(n-1),$$

where ${d}_{ij}$ is the shortcut distance between patentee i and j; n indicates the total number of patentees in the network.

We select the limit system indicator to measure the patentee's ability to use structural holes, that is, to measure whether there are structural holes around nodes. The lower the limit system of the patentee, the greater the bargaining chip of the patentee in GIN, thus the stronger the enterprise's ability to obtain information and knowledge in the network.

6 $${C}_{ij}={\left({p}_{ij}+\sum_{m}{p}_{im}{p}_{mj}\right)}^{2}.$$

${p}_{ij}$ represents the direct input of i to j; ${p}_{im}$ refers to the proportion of the relationship invested in m in the total patentee i, that is, the intensity ratio of the relationship between i and m; ${p}_{mj}$ represents the intensity ratio of m to j. We use 1-${C}_{ij}$ to represent the index of structural hole (*SH*).

Age of enterprise (*Age*) represents the duration of the enterprise. With the increase of enterprise operation time, the enterprise's knowledge and technical experience will continue to grow, and then affect the enterprise's technological innovation. By referring to Zhang^17^, the difference between the year studied by the enterprises in this paper and the year of their establishment is used to measure the technological innovation performance.

In the study of Gao et al.^15^, the number of patent applications in the first five years of the sample period was selected as the control variable. The patents created by R&D of enterprises include not only semiconductor patents, but also practical patents and other inventions. The R&D of these patents is achieved through the improvement of R&D and innovation capability of enterprises. Therefore, the inventions of other patents of enterprises will also affect the technological innovation of enterprises, and thus affect the invention of semiconductor patents of enterprises. In order to control the impact of semiconductor patents more specifically, this paper obtains the total number of patents of enterprises each year through the US patent database, and uses the quotient between the semiconductor patent holdings of enterprises in year t and the patent holdings of enterprises in year t to measure the percentage of semiconductor patents (*Percent*).

The study of Liu and Qi^39^ adopted the total asset scale of enterprises as the control variable. The increase of the total assets of semiconductor enterprises can increase the scale of business operation and help enterprises to strengthen research and development. Moreover, it is convenient for enterprises to increase their own scale, reduce market competitiveness and obtain the resources and research and development team of the acquired enterprises through M&A, so as to improve their own innovation. Therefore, this paper obtains the total asset data of the research samples from the annual financial statements of American stock exchanges and the official websites of each enterprise, which is used as the size of semiconductor enterprises (*Scale*).

As shown in Table 2, companies in the semiconductor industry have an average of 633 semiconductor patented inventions per year, which shows that the sample companies in the semiconductor industry have a strong overall innovation power. The average value of CC is 0.665, which shows that the enterprises in the network are largely out of the control of other enterprises, and the enterprises are less dependent on other enterprises in their innovation behavior, and their own innovation ability is strong. The average value of BC is 0.0298, which shows that the control ability of semiconductor companies in GIN is weak, and there are more direct links between patent rights in GIN. In terms of the structural hole in GIN, the mean value of SH is 0.722, which shows that the existence of structural holes in the network is very likely. The enterprise has an average of 42 years of business experience and learning experience. The proportion of semiconductor patents in the enterprise (Percent) shows that the overall innovation power of the enterprise is strong. In addition to semiconductor patents, many other patents have also been invented. The average asset size of semiconductor companies is 61.716 billion U.S. dollars.

**Table 2:** Date description.

Variable	Sample	Mean	Std	Min	Max
Patent	100	632.8	739.0	1	3631
CC	100	0.665	0.1067	0.4318	1
DC	100	0.472	0.199	0.105	1
BC	100	0.0298	0.0462	0	0.2601
SH	100	0.722	0.0622	0.500	0.811
SH^2^	100	0.526	0.085	0.25	0.657
Age	100	41.80	28.85	9	144
Percent	100	0.494	0.247	0.036	1
Scale	100	61.716	88.265	0.0124	406.794

## Empirical analysis

### Baseline regression

Table 3 shows the results of the empirical regression using the negative binomial regression model developed in this paper. Model (1) demonstrates the relationship between the predicted variable and the control variables, and the regression results significantly indicate that the choice of control variables is appropriate. Models (2), (3), (4), and (5) show the relationship between the predicted variable and the explanatory variables under the control variables, respectively.

**Table 3:** Results regression analysis.

Variables	(1)	(2)	(3)	(4)	(5)
	Patent	Patent	Patent	Patent	Patent
DC		1.380***			
		(0.411)			
CC			0.0314***		
			(0.00819)		
BC				0.0769***	
				(0.0239)	
SH					− 43.32*
					(24.50)
SH^2^					33.08*
					(17.78)
Age	0.010***	0.00677**	0.00592*	0.00551	0.00754**
	(3.10)	(0.00336)	(0.00328)	(0.00345)	(0.00345)
Percent	2.008***	1.977***	2.030***	2.070***	1.933***
	(6.14)	(0.298)	(0.294)	(0.305)	(0.313)
Scale	0.007***	0.00604***	0.00573***	0.00552***	0.00642***
	(5.76)	(0.00120)	(0.00116)	(0.00119)	(0.00123)
Constant	4.266***	3.822***	2.406***	4.286***	18.36**
	(19.69)	(0.235)	(0.518)	(0.203)	(8.456)
Observations	100	100	100	100	100

Standard errors are in parentheses, and *, ** and *** indicate significant at the 10%, 5% and 1% levels, respectively.

In model (2), it can be seen that after controlling for the variables of firm age and semiconductor patent share, the relationship between *DC* and the predicted variable shows a positive and significant effect, indicating that the *DC* of a firm in GIN has a positive and significant effect on the innovation of semiconductor firms, β = 1.38, p < 0.01, which means that the hypothesis H2 is supported. This indicates that patentees can improve their innovation performance by increasing their own and direct contact with other patentees, obtain more sources of information and resources channels, and learn explicit and tacit knowledge, thus enhancing corporate innovation technology and improving innovation performance. From the regression results of model (3), after controlling for the selected control variables, it is found that the *CC* of enterprises in the international innovation network has a positive and significant effect on the innovation performance of semiconductor enterprises, β = 0.0314, p < 0.01, which means that the hypothesis H4 is supported. This indicates that enterprises in the GIN network can reduce the distance to other enterprises, reduce the access to knowledge and information resources by shortening the cost, enhance the speed of information resource dissemination, and obtain information resources more easily, thus enhancing enterprise learning efficiency and reducing cost investment, and helping enterprises to improve their innovation technology. In model (4), the relationship between the *BC* of enterprises in GIN and the innovation performance of semiconductor enterprises is positive and significant, which supports the hypothesis H3, indicating that enterprises can obtain more resources, information and knowledge by controlling the access to resources of other enterprises, i.e. acting as an "intermediary". This can enhance the technological innovation capability of enterprises. In model (5), it is found that the *SH* in GIN shows a positive U-shaped relationship with the innovation performance of enterprises, and the coefficient of the squared term of the *SH* is significantly positive (β = 33.08, p < 0.1), so the hypothesis H1 is verified. This indicates that the knowledge, information and resources acquired by semiconductor companies in GINs vary according to their embedding position in the GIN. However, the knowledge, information and resources from "all directions" will lead to an increase in the cost and time efficiency of semiconductor companies in screening heterogeneous or tacit knowledge, information and resources, which will have a negative impact on or inhibit the innovation of companies. With the improvement of screening and learning ability, enterprises can quickly acquire, utilize and absorb the favorable information, knowledge and resources they have selected. With the enhanced ability to acquire resources, firms will eventually enhance their own innovation efficiency.

### Two-stage least squares regression

Firms with higher innovation capabilities have better knowledge, information, resources, which may attract other firms to establish partnerships with the firm, and thus enhance the position of semiconductor firms in the GIN, ultimately leading to an inverse causal relationship between the embeddedness of semiconductor firms in the GIN and innovation performance. In order to alleviate the endogeneity problem and refine the causal inference, a two-stage least squares regression with instrumental variables is used to implement the empirical analysis. For the use of instrumental variables, refer to the endogeneity treatment by Yin et al.^40^, the *CC* and *SH* are lagged by one period as the respective instrumental variables, and two-stage least squares regression analysis is performed, and the results are shown in Tables 4 and 5.

**Table 4:** First stage regression results.

Variables	(1)	(2)	(3)	(4)	(5)
	DC	CC	BC	SH	SH^2^
ivDC	0.761***				
	(11.36)				
ivCC		0.768***			
		(0.0632)			
ivBC			0.899***		
			(0.0599)		
ivSH				− 3.174**	− 5.040**
				(1.474)	(1.955)
ivSH^2^				2.732**	4.248***
				(1.086)	(1.441)
Control variables	Yes	Yes	Yes	Yes	Yes
Sample size	80	80	80	80	80
Adjusted ${R}^{2}$	0.727	0.757	0.838	0.395	0.423

Standard errors are in parentheses, and ** and *** indicate significant at the 5% and 1% levels, respectively.

**Table 5:** Second stage regression results.

Variables	(1)	(2)	(3)	(4)
	Patent	Patent	Patent	Patent
DC	2.108***			
	(0.0624)			
CC		0.045***		
		(0.0571)		
BC			0.098***	
			(0.1117)	
SH				− 65.94***
				(25.21)
SH^2^				52.03***
				(18.09)
Control variables	Yes	Yes	Yes	Yes
Sample size	80	80	80	80
Adjusted ${R}^{2}$	0.710	0.761	0.805	0.659

Standard errors are in parentheses, and *** indicate significant at the 1% levels, respectively.

In the first stage of the regression, each centrality indicator of semiconductor companies was regressed separately from the structural holes on their instrumental variables, and the results were significantly correlated. Therefore, the instrumental variables satisfy the condition of the existence of correlation with the alternate explanatory variables. In terms of testing for weak instrumental variables, the F-statistics of the regressions of centralities and the corresponding instrumental variables in the first stage are 129.122, 147.872, and 225.237, all of which are greater than 10, indicating that there are no weak instrumental variables in the model, and the CDW tests of structural holes and their instrumental variables also indicate that there are no weak instrumental variables. In the second stage regression, the number of semiconductor patents of the firms was regressed separately with the fitted values of each network centrality obtained from the first stage regression, and the results showed that all of them were significant at the 1% level, which was basically consistent with the above empirical results.

### Robustness tests

The results of the empirical analysis above show that the three centrality indicators in the global innovation networks formed through the undirected symmetric matrix of patent citation relationships among firms and thus constructed have positive effects with firm innovation performance, respectively, while the structural holes have a U-shaped impact relationship with firm innovation performance. To verify the reliability of these findings, we perform robustness tests by transforming the measured indicators of the predicted variable with a one-period lag of the explanatory variables.

The predicted variable (innovation performance of the firm) in the paper is measured by the number of semiconductor patent applications filed by the firm in year t. In this paper, by changing the measurement of the predicted variable, the total number of patent applications (*tpatents*) filed by the firm in year t is taken as a new measure of the firm's innovation performance in that year. The robustness regression results of the model are shown in Table 6.

**Table 6:** Robustness tests (with *tpantents*).

Variables	(1)	(2)	(3)	(4)
	tpatents	tpatents	tpatents	tpatents
DC	1.664***			
	(3.98)			
CC		0.035***		
		(0.00837)		
BC			0.0780***	
			(0.0244)	
SH				− 42.70*
				(22.95)
SH^2^				33.44**
				(16.73)
Control variables	Yes	Yes	Yes	Yes
Observations	100	100	100	100

Standard errors are in parentheses, and *, ** and *** indicate significant at the 10%, 5% and 1% levels, respectively.

In the robustness regression, the overall regression results are more significant after replacing the measurement of the predicted variable. The *DC* in the GIN showed a positive and significant effect with firm innovation performance (β = 2.089, p < 0.001), and the significance can still be verified. The *CC* and *BC* in the network also show positive effects with semiconductor firm innovativeness, respectively. Finally, the relationship between *SH* in the network and firm innovation performance also shows a positive U-shape.

The explanatory variables from 2014 to 2018 are treated with a one-period lag in the robustness regression shown in Table 7. The four models in Table 7 represent the negative binomial regression results of the three centrality indicators and the structural hole on the firm's innovation performance with a one-period lag, respectively. The regression results show that the sign of the coefficients of the three centrality indicators and the structural hole indicators remain the same and the empirical regression results are still significantly consistent, indicating that the one-period lag treatment has little effect on the stability of the model.

**Table 7:** Robustness test (with one lag period).

Variables	(1)	(2)	(3)	(4)
	tpatents	tpatents	tpatents	tpatents
DC	2.116***			
	(5.34)			
CC		0.0428***		
		(0.00780)		
BC			0.102***	
			(0.0251)	
SH				− 48.79**
				(23.47)
SH^2^				38.84**
				(17.00)
Control variables	Yes	Yes	Yes	Yes
Observations	80	80	80	80

Standard errors are in parentheses, and ** and *** indicate significant at the 5% and 1% levels, respectively.

### Heterogeneity analysis

#### Operation mode

As technology continues to advance, the semiconductor industry has become a high-tech industry and a more mature industry, and the monopolistic nature of the business has gradually emerged. In order to survive in the highly monopolistic environment, some companies have integrated their business and chosen different operation models. Different operation models have emerged in the semiconductor industry: integrated device manufacture (IDM), fabless mode, foundry mode. Among them, the first two models are the most common operating models in the industry. The IDM model represents the broadest scope of business and integrates multiple industry chains, enabling collaboration and optimization in semiconductor design, manufacturing and packaging to explore technological innovation, but with high capital consumption and management costs. Companies with the Fabless model focus on circuit design and sales segments, with low capital investment and lack of collaborative processes. Therefore, the operation mode may influence the technological innovation of a company through the amount of capital investment, operating cost and scale of technology development. In order to study the impact of operation mode and to verify the stability of the empirical findings, we analyze the firms in sub-samples according to the operation mode, and the results are shown in Tables 8 and 9.

**Table 8:** IDM mode.

Variables	(1)	(2)	(3)	(4)
	Patent	Patent	Patent	Patent
DC	0.474			
	(1.18)			
CC		0.0118		
		(0.00795)		
BC			0.0254	
			(0.0231)	
SH				− 41.96*
				(22.78)
SH^2^				30.62*
				(16.64)
Control variables	Yes	Yes	Yes	Yes
Observations	65	65	65	65

Standard errors are in parentheses, and * indicate significant at the 10% levels, respectively.

**Table 9:** Fabless mode.

Variables	(1)	(2)	(3)	(4)
	Patent	Patent	Patent	Patent
DC	3.306***			
	(3.82)			
CC		0.0828***		
		(0.0191)		
BC			0.234***	
			(0.0663)	
SH				− 50.92
				(42.94)
SH^2^				30.50
				(31.09)
Control variables	Yes	Yes	Yes	Yes
Observations	35	35	35	35

Standard errors are in parentheses, and *** indicate significant at the 1% levels, respectively.

The regression results show that the two models are different. The regression results for each indicator of centrality were significant in the Fabless model, but not in the regression of the IDM model. The structural hole, on the other hand, was significant in the IDM model regression and insignificant in the Fabless model regression. Overall, the regression results in the Fabless model are more consistent with the original regression results. This indicates that the difference in operation mode affects the effect of the embedding of GIN on the innovation performance of firms. Firms should choose the Fabless model to operate their own firms in order to obtain more efficient innovation power.

#### Regions

Referring to the paper by Liu and Qi^39^, the region of enterprises as a heterogeneous characteristic would make the innovation effect of network change. We classify the enterprises according to the region of headquarters into two different regions: Asia and development area (Europe and America). Since the cultural backgrounds of Europe and America differ greatly from those of Asia, and the economic development of Europe and America is better than that of Asia, and most of the highly developed technological inventions and research talents are concentrated in Europe and America, does the regional difference have a differential impact on the impact results of the tests? The empirical results are shown in Tables 10 and 11.

**Table 10:** Regression results for Europe and U.S.

Variables	(1)	(2)	(3)	(4)
	Patent	Patent	Patent	Patent
DC	4.159***			
	(7.21)			
CC		0.0891***		
		(0.0123)		
BC			0.302***	
			(0.0453)	
SH				− 14.45*
				(40.15)
SH^2^				17.44
				(28.79)
Control variables	Yes	Yes	Yes	Yes
Observations	65	65	65	65

Standard errors are in parentheses, and *, *** indicate significant at the 10% and 1% levels, respectively.

**Table 11:** Regression results for Asia.

Variables	(1)	(2)	(3)	(4)
	Patent	Patent	Patent	Patent
DC	0.0308			
	(0.559)			
CC		0.00722		
		(0.0100)		
BC			0.0145	
			(0.0240)	
SH				− 25.88
				(25.77)
SH^2^				17.24
				(19.03)
Control variables	Yes	Yes	Yes	Yes
Observations	35	35	35	35

Standard errors are in parentheses.

In the sub-sample regressions, the results for each embedding indicator in Europe and the United States are consistent with the overall regression results, while the results for Asia are not significant. This suggests that this regional variability has a significant impact on the innovation effect of GIN in semiconductor firms. The reason for this variability may be due to the different cultural and innovation environments in which firms are located.

In integrating our empirical findings with the theoretical framework of global innovation networks (GINs), we observe that the structural positions within these networks, as defined by our analyzed metrics, significantly impact the innovation performance of semiconductor companies.

Specifically, our data reveal a positive U-shaped relationship associated with structural holes, an insight that diverges from traditional linear expectations. This indicates that while initial increases in network structural holes may lead to confusion or information overload, beyond a certain threshold, the ability to span diverse informational voids contributes positively to innovation. This nuanced relationship underscores the complex dynamics within semiconductor industry innovation networks, where not just connectivity but the right type of connectivity determines innovation efficacy.

Moreover, the empirical manifestation of this *U*-shaped curve aligns with theories suggesting that excessive cohesiveness can stifle innovation by limiting novel information, whereas optimal structural holes provide unique access to diverse, non-redundant information. However, the semiconductor industry's high-tech context magnifies the significance of efficiently navigating these structural holes, emphasizing a balance between information diversity and overload.

The disparity between our findings and traditional views, particularly regarding the effects of network structures on innovation, adds a new layer of understanding to the GIN dynamics within the semiconductor sector. It illustrates the importance of network position not merely for information access but for effective information utilization and innovation generation.

Therefore, our study not only corroborates the vital role of GINs in shaping firms’ innovation outcomes but also highlights the nuanced mechanisms through which specific network structures like structural holes influence the complex process of technological innovation in the semiconductor industry.

## Conclusions

In extending our conclusions, we have taken into account the valuable feedback regarding the comparison of our research findings with existing studies in the realm of Global Innovation Networks (GIN) and their influence on corporate innovation performance, particularly within the semiconductor industry. Consistent with the studies by Meng^10^ and Wan^9^, our research corroborates the significant positive impact of a firm's network position, as indicated by metrics such as degree centrality, betweenness centrality, and closeness centrality, on its innovation performance. This finding underscores the importance of strategic positioning within GINs for semiconductor companies aiming to enhance their innovative capabilities. Furthermore, our identification of a positive U-shaped relationship between structural holes and innovation performance expands upon the insights provided by Yao and Gong^18^, suggesting a nuanced approach to leveraging network structures for innovation. While our study primarily uses patent applications as a proxy for innovation performance—a common approach in existing literature—we acknowledge the limitations this metric may present in capturing the full spectrum of corporate innovation. Future research could build on our findings by incorporating a broader range of innovation indicators, thereby enriching the understanding of GIN's role in fostering technological advancements within the semiconductor sector and beyond. This approach aligns with our commitment to contributing to the nuanced and evolving discourse on global innovation networks and their impact on industry-specific innovation dynamics.

Using social network analysis, we establish a GIN based on the undirected binary matrix through patent citation relationships between multinational semiconductor firms from 2014 to 2018. This study investigates how the firms' embeddedness in the GIN affects their innovation performance. Our findings suggest that: Firstly, degree centrality has a positive impact on semiconductor companies' innovation performance. By cooperating with other companies through patents, semiconductor firms can expand their scale range in the GIN and gain access to external knowledge, resources, and information. As a result, they can reduce the human, financial, material, and time investment required for R&D, thus enhancing their innovation capabilities.

Secondly, closeness centrality positively contributes to improving firms' innovation performance. By establishing direct patent partnerships with many other firms in GIN, semiconductor firms can reduce the distance to other firms' nodes, enabling them to acquire heterogeneous resources more efficiently and with higher quality, improving the efficiency of their innovation R&D.

Thirdly, betweenness centrality can promote semiconductor companies' innovation technology. By playing the role of "intermediaries" in GIN, companies can selectively transmit and obtain the information, knowledge, and resources disseminated and circulated by other firms in the network, enhancing their innovation capabilities.

Finally, there is a significant U-shaped relationship between the structural holes in GIN in which semiconductor firms are located and their innovation performance. The ability to use structural holes in GIN is strengthened as the lower the restriction of firms in the network, the more heterogeneous or complementary resources and information firms can obtain. However, when redundant information, homogeneous resources, and heterogeneous resources are disseminated at the same time, inhibiting the innovation and development of new technologies. For semiconductor firms, the structural holes they occupy have a positive U-shaped effect on their innovation performance, as they can use the circulating heterogeneous resources to expand their knowledge reserve and promote their innovation.

In light of our investigation into the overarching network attributes and specific network configurations within the Global Innovation Network (GIN), alongside empirical analysis, we tender the following refined guidance for industry practitioners.

It's imperative for companies to recognize the nuanced function that structural holes within GIN play. The identified positive "U" shaped correlation between structural holes and innovation output underlines the necessity for firms to strategically leverage these voids for accessing diverse resources, whilst cautiously avoiding an excessive dependency on them. Firms are encouraged to cultivate robust mechanisms for vetting and selecting resources, aiming to differentiate between those of high and low quality. Enhancing organizational capabilities in learning and assimilation will serve to bolster innovation performance over the long term, enabling firms to navigate the complex landscape of global innovation more adeptly.

Moreover, the enhancement of network management competencies emerges as a critical strategy for ensuring the optimal exploitation of networked resources. Establishing a comprehensive patent management framework, alongside reinforcing intellectual property rights, becomes essential in safeguarding the integrity and efficacy of network-derived resources. Concurrently, fostering an ecosystem that encourages effective communication and reciprocal learning among network entities can significantly amplify the flow and utility of knowledge and information across the GIN.

Thus, our empirical findings underscore the strategic importance of network position and structure in augmenting innovation performance. By meticulously managing their position within the GIN—paying particular attention to the dynamics of degree centrality, betweenness centrality, closeness centrality, and structural holes—firms can harness the full potential of their network to fuel sustainable innovation and competitive advantage.

In addition, future research could consider use different database, like the JPO and EPO databases, and investigate other industries to observe the impact of GIN.

## Data Availability

The data that support the findings of this study are available from the corresponding author upon reasonable request.
